# Dynamic Mechanical Characterization of Warm-Mixed Steel Slag-Crumb Rubber Modified Asphalt Mixture in Wide- and Narrow-Frequency Domains

**DOI:** 10.3390/polym17111449

**Published:** 2025-05-23

**Authors:** Fei Zhang, Bingyuan Huo, Chao Li, Heng Liu, Pengzhi Li, Yongming Xing, Lan Wang, Pucun Bai

**Affiliations:** 1 School of Materials Science and Engineering, Inner Mongolia University of Technology, Hohhot 010051, China; zhangfei@imut.edu.cn; 2 School of Civil Engineering, Inner Mongolia University of Technology, Hohhot 010051, China; h1606864536@163.com (B.H.); nmggydx@21cn.com (C.L.); 18748166534@163.com (P.L.); 3 Inner Mongolia Autonomous Region Key Laboratory of Green Construction and Intelligent Operation and Maintenance of Civil Engineering, Hohhot 010051, China; xym@imut.edu.cn; 4 Inner Mongolia Autonomous Region Institute of Transportation Science and Development, Hohhot 010051, China; 15147136841@163.com

**Keywords:** road engineering, warm-mixed steel slag-crumb rubber modified asphalt mixture, dynamic modulus, 2S2P1D model, MHN model

## Abstract

To investigate the dynamic mechanical properties of warm-mix steel slag-crumb rubber modified asphalt mixtures across wide- and narrow-frequency domains and evaluate the applicability of warm-mix technology, four distinct mixtures were prepared. The dynamic modulus characteristics under measured temperatures and frequencies were initially analyzed through complex modulus testing to elucidate narrow-frequency-domain mechanical behavior. Subsequently, leveraging the linear viscoelastic (LVE) theory and time–temperature superposition principle (TTSP), both the 2 Springs, 2 Parabolic Elements and 1 Dashpot (2S2P1D) mechanical element model and Modified Havriliak–Negami (MHN) mathematical model were established based on experimental data to characterize wide-frequency-domain dynamic responses. The results demonstrate substantial consistency in mechanical interpretation between narrow- and wide-frequency domain datasets, with enhanced information resolution achieved in wide-frequency analysis. Both models demonstrate comparable accuracy in characterizing the thermomechanical behavior of warm-mix steel slag-crumb rubber modified asphalt mixture across extended frequency and temperature ranges, while showing negligible performance discrepancies between the 2S2P1D and MHN formulations. Furthermore, both Cole–Cole and Black diagrams convincingly demonstrate the reliability of model predictions. This systematic investigation confirms the technical viability of warm-mix steel slag-crumb rubber modified asphalt mixture while establishing a dual-validated modeling framework for comprehensive performance prediction.

## 1. Introduction

The current global challenges of environmental pollution and resource scarcity remain critical, driving the road construction industry to prioritize circular economy implementation. Steel slag, an industrial byproduct from steel manufacturing, presents cost advantages over natural aggregates while enhancing asphalt mixture performance through improved energy efficiency and environmental sustainability [1]. Concurrently, waste tire recycling through crumb rubber production enables the development of high-performance modified asphalt binders. The synergistic utilization of steel slag aggregates with crumb rubber modified asphalt binders addresses multiple sustainability objectives in pavement engineering. However, due to the high viscosity of crumb rubber modified asphalt, the production and construction of high-temperature requirements, high energy consumption and generate a large amount of dust and exhaust, such method is contrary to the concept of Chinese’s green development, but the application of warm-mixing technology to make up for this shortcoming. In the high-temperature environment, asphalt pavement is subjected to repeated vehicle loading, it is easy to produce rutting disease; in the medium and low temperature environment, asphalt pavement is subjected to repeated vehicle loading and temperature stress, it is easy to produce low-temperature cracking disease and fatigue cracking disease. These diseases will seriously affect the service life of the road and traffic safety. An extensive review of existing literature reveals that substantial progress has been achieved in the research domain of steel slag asphalt mixtures. To establish a rigorous cross-comparison framework, we have developed a standardized comparative matrix (Table 1) that methodically catalogs seminal domestic and international findings, with critical parameters including mixture gradation, slag substitution ratios, and mechanical performance explicitly highlighted.

Current research advancements in steel slag asphalt mixtures demonstrate partial success, yet reveal critical knowledge gaps in systematic characterization. While existing studies have validated steel slag’s feasibility as aggregate replacement and established basic performance parameters of warm-mix steel slag asphalt, the research landscape remains fragmented. Critical limitations persist in three aspects: (a) Overemphasis on macroscopic pavement properties without sufficient micromechanical analysis; (b) Narrow-frequency domain investigations lacking broad-spectrum viscoelastic characterization; (c) Insufficient integration of advanced modification techniques with warm-mixing techniques. In addition, asphalt pavements are mainly affected by dynamic loading during service, and asphalt material is a typical viscoelastic material, so viscoelastic behavior under dynamic loading is crucial to understand the pavement performance. Typically, asphalt mixtures exhibit LVE properties under small strain levels or limited cyclic loading [14]. The complex modulus test under oscillatory loading is the most traditional method to study the LVE of asphalt mixtures. It allows obtaining the dynamic modulus and phase angle of asphalt mixtures in the LVE range. This study comprehensively characterizes the dynamic mechanical behavior of steel slag asphalt mixtures through systematic complex modulus testing across extended frequency ranges and temperature spectra, combined with LVE theoretical analysis. The research establishes frequency-temperature superposition master curves, providing critical insights for optimizing both narrow-frequency domain (short-term performance) and broad frequency domain (long-term performance) characteristics in steel slag-crumb rubber modified asphalt mixture.

In addition, this study also presents a novel investigation into the dynamic viscoelastic behavior of warm-mix steel slag-crumb rubber modified asphalt mixtures through comprehensive narrow and broad frequency domain analyses, significantly advancing knowledge in sustainable pavement materials. The research innovatively integrates three eco-friendly technologies—steel slag recycling, waste tire rubber powder utilization, and warm-mix technology—while establishing dual validation through advanced rheological modeling (2S2P1D and MHN). The developed modeling framework not only provides engineers with a robust tool for designing durable, low-carbon pavements but also establishes a critical reference framework for subsequent investigations into dynamic mechanical characteristics of other composite materials. This integrated approach demonstrates both theoretical sophistication and practical applicability, effectively bridging material science innovation with sustainable infrastructure development requirements.

## 2. Materials and Methods

### 2.1. Materials

#### 2.1.1. Crumb Rubber Modified Asphalt

Crumb rubber modified asphalt binder is prepared from virgin asphalt and crumb rubber according to the specific process. The performance grade of virgin asphalt is PG 58-22. Crumb rubber can be produced by mechanical grinding at room temperature. In addition, all of the crumb rubber was prepared from scrap bias tires from the same source. The virgin asphalt was purchased from Inner Mongolia Luda Asphalt Co., Ltd., (Hohhot China) while the crumb rubber was purchased from the Hubei Anjie Road & Bridge Technology Co., Ltd. (Ezhou City, Hubei Province, China).The crumb rubber modified asphalt binder was prepared by blending 20% of 60-mesh crumb rubber by the weight of virgin asphalt at 180 °C and 700 rpm/min using a high speed open blade mixer (FLUKO, Shanghai, China). And technical parameters of crumb rubber modified asphalt are shown in Table 2. The table demonstrates that all technical parameters of crumb rubber modified asphalt comply with the specification requirements.

#### 2.1.2. Warm Mix Additives

Warm-mix crumb rubber modified asphalt binder is prepared by adding warm-mix additive to the crumb modified asphalt binder. Warm-mix additive (SDYK) is surfactant, purchased from Wuxi Daoruide Chemical Technology Co. (Wuxi, Jiangsu Province, China). The content was 1% by weight of the asphalt binder (recommended by the manufacturer). It was incorporated into the asphalt binder by mixing at 180 °C and 500 rpm for half an hour using a conventional mechanical mixer (FLUKO, Shanghai, China). And the technical parameters of the warm-mix additives are shown in Table 3. The table demonstrates that all technical parameters of the warm-mix additive comply with the specification requirements.

#### 2.1.3. Aggregates and Filler

The aggregates selected in this study are basalt and steel slag, in which basalt aggregate was purchased from a quarry located in Zhuozishan, Inner Mongolia Autonomous Region, China, and the specifications of the aggregate are 0–3 mm, 3–5 mm, 5–10 mm and 10–20 mm. The steel slag aggregate from Baotou Iron and Steel Group Co., Ltd. (Baotou, Inner Mongolia Autonomous Region, China). belongs to hot smothered steel slag, and its original specifications are only 0–10 mm, 10–20 mm two grades, in order to keep the same specification with basalt aggregate, it was screened by motorized sieve machine, and four specifications were obtained, which are 0–3 mm, 3–5 mm, 5–10 mm, 10–20 mm four grades. The technical parameters of basalt aggregate and steel slag aggregate all meet the requirements of JTG E42-2005 [15]. The technical specifications of coarse and fine aggregates for both steel slag and basalt are presented in Table A1 and Table A2 of Appendix A.1, respectively. The limestone mineral filler, produced through mechanical pulverization of high-calcium carbonate rock, exhibited critical physical characteristics including a specific gravity, plasticity index (PI), and particle size distribution—all parameters demonstrating full compliance with JTG E42-2005 [15]. specifications for highway construction materials. The technical specifications of limestone mineral filler are presented in Table A3 of Appendix A.1.

### 2.2. Methods

#### 2.2.1. Methods for Grading Design of Steel Slag Crumb Rubber Modified Asphalt Mixtures

**Figure 1:**
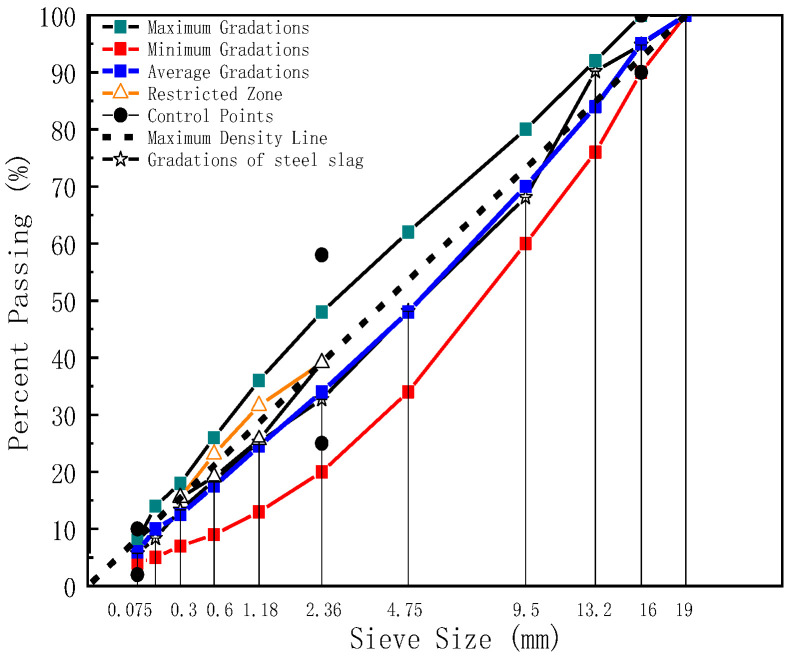
The gradation of steel slag.

**Figure 2:**
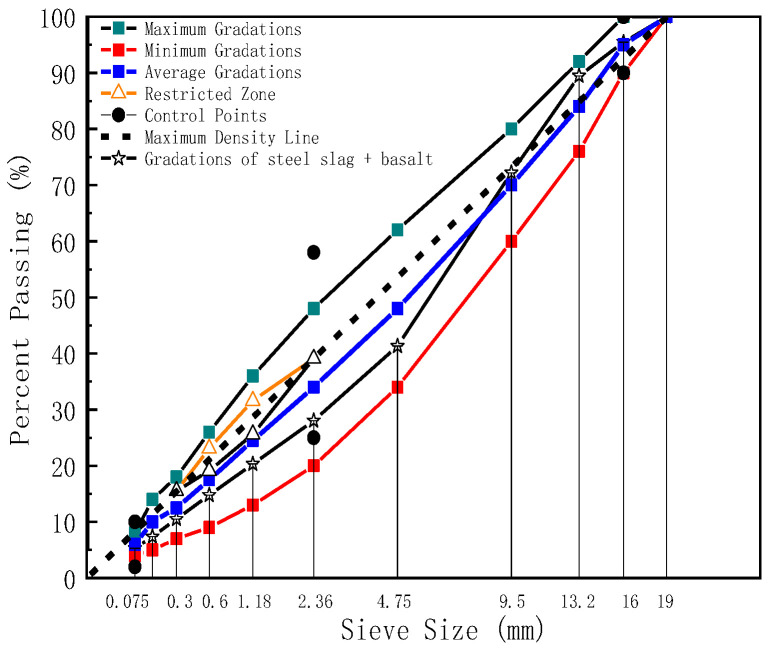
The gradation of steel slag & basalt.

Dense graded (AC-16) was utilized in the following text. In the design process of aggregate blend percentages, one type of aggregate skeleton consists of steel slag aggregate only; the other type consists of steel slag aggregate and basalt aggregate together, in which the steel slag portion consists of three grades of 3–5 mm, 5–10 mm, and 10–20 mm, and the basalt part only includes 0~3 mm. Moreover, for any grade of aggregate in the four specifications, three repeated sieving experiments were tested to reduce the error, the range of gradation was given and the synthetic gradation was controlled using the planning solving method. Table 4 shows different aggregate stockpiles blended by the percentages, and Figure 1 and Figure 2 show the gradation of the steel slag aggregates and steel slag & basalt aggregates.

The asphalt binders used in this paper are all crumb rubber modified asphalt binders. There are two types of aggregate, one is composed of steel slag aggregate only; the other is composed of steel slag aggregate and basalt aggregate together. Considering the research requirement, the corresponding warm-mix asphalt mixture was formed by introducing the warm-mix technology on the basis of the conventional hot mix mixture. As a result of the previous analysis, four different asphalt mixtures, namely, warm-mix steel slag-crumb rubber modified asphalt mixture (WSS), hot mix steel slag-crumb rubber modified asphalt mixture (HSS), warm-mix basalt-steel slag hybrid crumb rubber modified asphalt mixture (WB), and hot mix basalt-steel slag hybrid crumb rubber modified asphalt mixture (HB), were selected to complete the study. The optimum asphalt content and the corresponding volume parameters of warm and hot mix steel slag-crumb rubber modified asphalt mixtures were calculated based on the Marshall test method. The results of volume parameters at optimum asphalt content are shown in Table 5. A rigorous three-sample protocol was implemented to ensure measurement reliability, with three independent replicate tests conducted for each mixture and a statistical control criterion enforced requiring all experimental measurements to remain within 1.15 standard deviations of the mean.

#### 2.2.2. Methods for Specimen Fabrication of Complex Modulus Test

Based on volumetric parameters determined through Marshall testing and mix temperature considerations, cylindrical specimens were initially fabricated using Texler 5850 Superpave gyratory compactor (Troxler, Raleigh, NC, USA) to achieve standard dimensions of 150 mm in diameter × 170 mm in height. Subsequently, core drilling and precision cutting techniques were employed to prepare derived specimens measuring 100 mm in diameter × 150 mm in height specifically for complex modulus characterization. This two-stage fabrication process ensured dimensional compliance with respective testing protocols while maintaining material continuity between original and modified specimen configurations.

#### 2.2.3. Method for Complex Modulus Test of Asphalt Mixtures

The test equipment was closed-loop servo-hydraulic universal testing machine (UTM-100) manufactured by IPC Australia, which consisted of test software, control and data acquisition system, loading frame, hydraulic pump, hydraulic diverter, temperature control box and test components. In this paper, the uniaxial compression complex modulus test was conducted in accordance with the test method described in the AASHTO TP 79-15 [16]. The axial strain was monitored by three linear variable differential transducers (LVDT) with a gauge length of 70 mm, which were mounted with 120 degree around the middle side of specimens. The average of three LVDT was retained as the axial strain. The load level was auto-adjusted to ensure that the maximum axial strain no more than 70 με, which was within the LVE domain [17]. Besides, the load was monitored by the load cell installed on the actuator. The temperature-controlled box is heated up to the test temperature, and the specimen is placed in the box to keep warm for 4~5 h, in which 5 °C is kept warm for more than 8 h, so that the inside of the specimen reaches the test temperature. At the beginning of the test, the actuator of the system was lowered to make slight contact with the loading plate on the specimen, and the three LVDT in the UTM-100 system were observed to be working properly, the zeroing operation was performed, the test load was applied, and the specimen was pre-pressurized with 5% of the contact load for 10 s to ensure that the specimen was in good contact with the upper and lower loading plates.

The experimental protocol requires sequential testing progression from low- to high-temperature regimes, with each temperature stage incorporating frequency sweeps from high to low ranges. A critical termination criterion was established wherein test specimens must be discarded upon exceeding cumulative plastic deformation thresholds of 1500 με during individual frequency-stage evaluations at designated temperatures. The test temperatures were 5 °C, 20 °C, 40 °C, 50 °C and 60 °C, and the test frequencies were 0.1 Hz, 0.5 Hz, 1 Hz, 5 Hz, 10 Hz and 25 Hz, respectively. Similar to the Marshall test results, during the complex modulus testing we implemented a rigorous three-sample protocol. For each mixture formulation, three independent replicate tests were conducted with statistical control criteria requiring all experimental measurements to remain within 1.15 standard deviations of the mean value.

## 3. Theoretical Background, 2S2P1D Model and MHN Model

The entire process of the complex modulus test has been described previously. in addition, the complex modulus test is the most commonly used method for characterizing the LVE behavior of asphalt mixture [18], which involves applying the uniaxial sinusoidal stress $\sigma_{0}e^{i\omega t}$ to a cylindrical specimen and measuring the resulting strain response $\varepsilon_{0}e^{i\left(\omega t-\varphi \right)}$. Thus, the complex modulus can be obtained from the ratio of the input stress to the output strain resultant, as computed in the Equation (1). The dynamic modulus is the absolute value of the complex modulus. It can be calculated by the ratio of amplitude stress to amplitude strain, as shown in Equation (2). The phase angle reflects the time lagging between stress and strain [19]. It can be calculated by the last five loading cycles. The function form can be obtained following the Equation (3).

(1) $$G^{*}=\frac{\sigma_{0}e^{i\omega t}}{\varepsilon_{0}e^{i\left(\omega t-\varphi \right)}}=\frac{\sigma_{0}}{\varepsilon_{0}}e^{i\varphi }=\frac{\sigma_{0}}{\varepsilon_{0}}\left(\cos\varphi +i\sin\varphi \right)=G^{\prime }+iG^{\prime \prime }$$

(2) $$\left|G^{*}\right|=\sqrt{{\left(G^{\prime }\right)}^{2}+{\left(G^{\prime \prime }\right)}^{2}}=\frac{\sigma_{0}}{\varepsilon_{0}}$$

(3) $$\varphi =\frac{t_{i}}{t_{p}}\times 360$$

where: $G^{*}$ is the value of complex modulus, MPa; $\sigma_{0}$ is the axial stress amplitude measured by load cell installed on the actuator, MPa; $\varepsilon_{0}$ is the axial strain amplitude measured by LVDT; i is the imaginary unit defined by $i^{2}=-1$; $G^{\prime }$ is the value of storage modulus, MPa; $G^{\prime \prime }$ is the value of loss modulus, MPa. $\left|G^{*}\right|$ is the value of dynamic modulus, MPa; φ is the phase angle, °; $t_{i}$ is the average time lagging of the last five loading cycles between stress and strain, s; $t_{p}$ is the average time of the last five stress cycles, s.

As viscoelastic materials, the mechanical behavior of asphalt mixtures changes significantly with temperature, loading frequency and time [20]. To ensure the durability of pavements under complex environmental conditions (e.g., high-temperature rutting, low-temperature cracking, and dynamic fatigue loading), rheological models must accurately capture their viscoelastic characteristics, especially in dynamic loading. However, conventional models (e.g., Sigmoidal and Burgers models) are often unable to describe these complex behaviors in a comprehensive manner, thus necessitating the use of more advanced rheological models. Given that mechanical element-based models and mathematical models are the most widely used in characterizing the viscoelasticity of asphalt mixtures, in this study, the best model from these two types of models was selected to study the broad-frequency rheological properties of steel slag-crumb rubber modified asphalt mixtures. For the above reasons, 2S2P1D mechanical element model and MHN mathematical model are developed in this paper to analyze the dynamic mechanical properties of steel slag-crumb rubber modified asphalt mixtures in the wide-frequency domain.

### 3.1. 2S2P1D Mode

The 2S2P1D model [21] is a generalized model derived from the Huet-Sayegh model. The model consists of two elastic elements, two parabolic elements and one dashpot element, which can accurately describe the rheological properties of asphalt binders and asphalt mixtures. According to Olard and Di Benedetto [12], the parabolic element is an analogous model with a parabolic creep function as shown in Equation (4).

(4) $$J\left(t\right)=\alpha {\left(\frac{t}{\tau }\right)}^{h}$$

where $J\left(t\right)$ is the creep compliance of the parabolic element, MPa^−1^; *h* is the constant related to the material properties, generally 0 < *h* < 1, when *h* = 0 for purely elastic materials, when *h* = 1 for purely viscous materials; α=1/λ is a dimensionless constant, λ is the relaxation time of the material; *t* is the loading time, s; τ is the characteristic time, s whose value depends only on the temperature.

Based on Equation (4) the complex modulus form of this parabolic element is known as Equation (5).

(5) $$G^{*}\left(\omega \right)=\frac{{\left(i\omega \tau \right)}^{h}}{\alpha \Gamma \left(h+1\right)}$$

where $G^{*}\left(\omega \right)$ is the complex modulus of the parabolic element, MPa; Γ is the gamma function; ω is the angular frequency of loading, rad/s.

The 7 parameter ($G_{0}$, $G_{g}$, μ, k, h, $\tau_{0}$, β) 2S2P1D viscoelastic constitutive model can be obtained by combining the above 2 parabolic elements with 2 elastic elements and one dashpot element. And the constitutive model of 2S2P1D is shown in Equation (6).

(6) $$G^{*}\left(\omega \right)=G_{0}+\frac{G_{g}-G_{0}}{1+\mu {\left(i\omega \tau \right)}^{-k}+{\left(i\omega \tau \right)}^{-h}+{\left(i\omega \beta \tau \right)}^{-1}}$$

where, $G_{0}$ is the static or equilibrium modulus when the frequency ω tends to very small value (zero), MPa; $G_{g}$ is the glass or transient modulus when the frequency ω tends to very large value (infinity), MPa; μ is a dimensionless model parameter, the value of which is related to the slope of the dynamic modulus master curve $\left|G^{*}\right|$ at low temperature/high frequency and the height of the peak point of the Cole–Cole plot; *k* and *h* are constant related to the material properties and 0 < *k* < *h* < 1, with *k* denoting the slope of the loss modulus $G^{\prime \prime }$ at the high value of the Cole–Cole diagram, and *h* denoting the slope of the loss modulus $G^{\prime \prime }$ at the low value of the Cole–Cole diagram; β is defined by the following Equation (7), and its value is related to the slope of the dynamic modulus master curve $\left|G^{*}\right|$ at high temperature/low frequency, and the larger the value of β, the larger the values of η and $\left|G^{*}\right|$.

(7) $$\beta =\frac{\eta }{\left(G_{g}-G_{0}\right)\tau }$$

where η is the Newtonian viscosity, Pa·s; τ is the characteristic time, and the value can be calculated by Equation (8), s; The parameter β of Equation (7) is temperature-dependent and can be calculated by shift factor equations within the temperature range usually tested, in this paper the WLF (Williams-Landel-Ferry) shift factor equation is used for the study and the equation is shown in Equation (9).

(8) $$\tau =\tau_{0}\times \alpha_{T}\left(T\right)$$

(9) $$\log\alpha_{T}\left(T\right)=\frac{-C_{1}\left(T-T_{r}\right)}{C_{2}+\left(T-T_{r}\right)}$$

where $\tau_{0}$ is the characteristic time of the asphalt mixture at the reference temperature $T_{r}$, the value of which can be determined directly during model fitting, s; $\alpha_{T}\left(T\right)$ is the time–temperature shift factor for any test temperature *T*; T is test temperature, °C; $T_{r}$ is the reference temperature, °C; $C_{1}$ and $C_{2}$ are constants determined by the thermodynamic properties of the material and are used as the fitting parameters in practical applications.

In this case, 40 °C was used as the reference temperature of 2S2P1D model.

### 3.2. MHN Model

Considering that the process of describing the viscoelastic behavior of asphalt mixtures using the Havriliak–Negami (HN) model exists that the modulus results obtained at high frequencies are located on the left side of the Cole–Cole plot, which is contrary to what is normally expected for asphalt concrete [22]. Therefore, the MHN model proposed by Tschoegl [23] was used in this study to model the dynamic modulus of steel slag-crumb rubber modified asphalt mixtures. In this case, the MHN model is shown in Equation (10).

(10) $$G^{*}\left(w_{r}\right)=G_{0}+\frac{G_{g}-G_{0}}{{\left[1+{\left(\frac{w_{0}}{iw_{r}}\right)}^{\alpha }\right]}^{\beta }}$$

where, $G_{0}$ is the static or equilibrium modulus when the reduced angular frequency $\omega_{r}$ tends to 0, MPa; $G_{g}$ is the glass or transient modulus when $\omega_{r}$ tends to infinity, MPa; $\omega_{0}$ is relates to the horizontal position of the storage modulus or loss modulus along the frequency axis; $\omega_{r}$ is reduced angular frequency, rad/s; *α* and *β* are model fitting parameters that control the width and skewness of the loss peaks, respectively.

Reduced angular frequency $\omega_{r}$ can be calculated by Equation (11). Similar to 2S2P1D model, the WLF equation is also used here for the study. Where the expression of the WLF equation is shown in Equation (9).

(11) $$\omega_{r}=\omega_{T}\times \alpha_{T}\left(T\right)$$

where, $\omega_{T}$ is the angular frequency at any temperature, rad/s; and when the test temperature $T=T_{r}$, then $\omega =\omega_{r}$.

Similar to the 2S2P1D model, 40 °C was also used as the reference temperature of MHN model.

## 4. Calibration of Model Parameters

The basic equations of the 2S2P1D and MHN models are described in the previous sections, but the calibration methods and parametric results of these models remain undiscussed. The calibration of rheological model parameters is the core of rheological research, which is an optimization process oriented by experimental data, and at the same time, this process needs to ensure the theoretical soundness of the model constitutive framework and the reliability of engineering predictions. In addition, accurate parameter calibration of the model is the foundation of intelligent material design and accurate management of pavement service life. The calibration procedures and quantitative parameters for the 2S2P1D and MHN models are presented as follows.

### 4.1. Calibration the Model Parameters of 2S2P1D Model

Applying De Moivre’s theorem and combining it with the results of Euler’s equations, an equivalent form of the 2S2P1D model can be derived as shown in Equation (12).

(12) $$G^{*}\left(\omega \right)=\frac{G_{g}-G_{0}}{1+A\left(\omega \right)+iB\left(\omega \right)}$$

where $A\left(\omega \right)$ and $B\left(\omega \right)$ can be represented by the following Equations (13) and (14), respectively.

(13) $$A\left(\omega \right)=\mu {\left(\omega \tau \right)}^{-k}\times \cos\left(\frac{k\pi }{2}\right)+{\left(\omega \tau \right)}^{-h}\times \cos\left(\frac{h\pi }{2}\right)$$

(14) $$A\left(\omega \right)=-\mu {\left(\omega \tau \right)}^{-k}\times \sin\left(\frac{k\pi }{2}\right)-{\left(\omega \tau \right)}^{-h}\times \sin\left(\frac{h\pi }{2}\right)-{\left(\omega \beta \tau \right)}^{-1}$$

By separating the real and imaginary parts of Equation (12), the corresponding storage and loss modulus can be obtained with the expressions shown in Equations (15)–(19).

(15) $$\begin{array}{l} G^{*}\left(\omega \right)=\frac{G_{g}-G_{0}}{1+A\left(\omega \right)+iB\left(\omega \right)}=G_{0}+\frac{\left(G_{g}-G_{0}\right)\times \left(1+A\left(\omega \right)-iB\left(\omega \right)\right)}{{\left(1+A\left(\omega \right)\right)}^{2}+B{\left(\omega \right)}^{2}}\\ =\left[G_{0}+\frac{\left(G_{g}-G_{0}\right)\times \left(1+A\left(\omega \right)\right)}{{\left(1+A\left(\omega \right)\right)}^{2}+B{\left(\omega \right)}^{2}}\right]+i\left[\frac{\left(G_{g}-G_{0}\right)\times \left(-B\left(\omega \right)\right)}{{\left(1+A\left(\omega \right)\right)}^{2}+B{\left(\omega \right)}^{2}}\right]=G^{\prime }\left(\omega \right)+G^{\prime \prime }\left(\omega \right) \end{array}$$

(16) $$G^{\prime }\left(\omega \right)=G_{0}+\frac{\left(G_{g}-G_{0}\right)\times \left(1+A\left(\omega \right)\right)}{{\left(1+A\left(\omega \right)\right)}^{2}+B{\left(\omega \right)}^{2}}=G_{0}+\frac{\Delta G\times \left(1+A\left(\omega \right)\right)}{DEN}$$

(17) $$G^{\prime \prime }\left(\omega \right)=\frac{\left(G_{g}-G_{0}\right)\times \left(-B\left(\omega \right)\right)}{{\left(1+A\left(\omega \right)\right)}^{2}+B{\left(\omega \right)}^{2}}=\frac{\Delta G\times \left(-B\left(\omega \right)\right)}{DEN}$$

(18) $$\Delta G=\left(G_{g}-G_{0}\right)$$

(19) $$DEN={\left(1+A\left(\omega \right)\right)}^{2}+B{\left(\omega \right)}^{2}$$

Combining the results of storage modulus and loss modulus, the dynamic modulus and phase angle results expressed in terms of the 2S2P1D model can be obtained, where the results of dynamic modulus are shown in Equation (20), and the results of phase angle are shown in Equation (21).

(20) $$\begin{array}{l} \left|G^{*}\left(\omega \right)\right|=\sqrt{{\left[G^{\prime }\left(\omega \right)\right]}^{2}+{\left[G^{\prime \prime }\left(\omega \right)\right]}^{2}}=\sqrt{{\left[G_{0}+\frac{\Delta G\times \left(1+A\left(\omega \right)\right)}{DEN}\right]}^{2}+{\left[\frac{\Delta G\times \left(-B\left(\omega \right)\right)}{DEN}\right]}^{2}}\\ =\sqrt{G_{0}^{2}+\frac{\Delta G\times \left(2G_{0}\times \left(1+A\left(\omega \right)\right)+\Delta G\right)}{DEN}} \end{array}$$

(21) $$\delta =\arctan\left(\frac{G^{\prime \prime }\left(\omega \right)}{G^{\prime }\left(\omega \right)}\right)=\arctan\left(\frac{\Delta G\times \left(-B\left(\omega \right)\right)}{\Delta G\times \left(1+A\left(\omega \right)\right)+G_{0}\times DEN}\right)$$

In order to obtain the model parameters, the parameter solution can be accomplished by constructing the error function $ef_{1}$ for the storage modulus and loss modulus, where the expression of the error function $ef_{1}$ is shown in Equation (22).

(22) $$ef_{1}=ef_{G^{\prime }}+ef_{G^{\prime \prime }}=\frac{1}{N}\sqrt{\sum_{i=1}^{N}{\left(\frac{{\left|G^{\prime }\left(\omega \right)\right|}_{m,i}-{\left|G^{\prime }\left(\omega \right)\right|}_{p,i}}{{\left|G^{\prime }\left(\omega \right)\right|}_{m,i}}\right)}^{2}}+\frac{1}{N}\sqrt{\sum_{i=1}^{N}{\left(\frac{{\left|G^{\prime \prime }\left(\omega \right)\right|}_{m,i}-{\left|G^{\prime \prime }\left(\omega \right)\right|}_{p,i}}{{\left|G^{\prime \prime }\left(\omega \right)\right|}_{m,i}}\right)}^{2}}$$

where: $ef_{1}$ is the error function of storage modulus and loss modulus, %; $ef_{G^{\prime }}$ is the error function of storage modulus, %; $ef_{G^{\prime \prime }}$ is the error function of loss modulus, %; N is the test sample point of storage modulus, which has the value of 30; $G^{\prime }{\left(\omega \right)}_{m,i}$ is the sample point of measured storage modulus, MPa; $G^{\prime }{\left(\omega \right)}_{p,i}$ is the sample point of storage modulus predicted from the 2S2P1D model, MPa; $G^{\prime \prime }{\left(\omega \right)}_{m,i}$ is the sample point of measured loss modulus, MPa; $G^{\prime \prime }{\left(\omega \right)}_{p,i}$ is the sample point of predicted from the 2S2P1D model, MPa.

Functional forms of storage modulus and loss modulus master curves of different steel slag-crumb rubber modified asphalt mixtures were constructed based on the 2S2P1D model. The fitting parameters of the model were calibrated by the least squares method using the WLF shifting technique, taking into account the measured results of storage modulus and loss modulus. Among them, the 2S2P1D model storage modulus master curve model has 7 unknown parameters ($G_{0}$, $G_{g}$, μ, k, h, $\tau_{0}$, β), in addition to 2 shift factor parameters ($C_{1}$, $C_{2}$) need to be determined. All model parameters in Equations (6) and (9) can be solved by constructing and minimizing the error function $ef_{1}$ for the storage and loss modulus. Where the expression of the error function $ef_{1}$ is given in Equation (22). All model parameters and shift factor parameters can be obtained using the Solve function of Microsoft Excel [24]. Finally, Table 6 compiles all 2S2P1D model parameter results (model parameters and shift factor parameters), demonstrating close agreement between our model fitting outcomes and those reported by Han [25] et al. Furthermore, Table 7 presents validation statistics associated with the model fitting process, all of which conclusively demonstrate the reliability of the models. Supplementary validation provided in Appendix A.2, Table A4 confirms that the goodness-of-fit criteria satisfy the “Excellent” classification standard.

### 4.2. Calibration the Model Parameters of MHN Model

Havriliak and Negami separated the real and imaginary parts in Equation (10) by successively applying De Moivere’s theorem to the denominator to extract the complex roots and then rationalizing the denominator to further obtain the analytic forms of the real and imaginary parts of the complex modulus based on the MHN model. The real component corresponds to the storage modulus, while the imaginary component represents the loss modulus. The specific expressions for these modulus characterized by the MHN model are presented in Equations (23)–(25).

(23) $$G^{\prime }\left(w\right)=G_{0}+(G_{g}-G_{0})\frac{{\left(\frac{w}{w_{0}}\right)}^{\frac{\alpha \beta }{2}}\cos\left[\beta \theta \left(w\right)\right]}{{\left[{\left(\frac{w}{w_{0}}\right)}^{\alpha }+2\cos\left(\frac{\alpha \pi }{2}\right)+{\left(\frac{w_{0}}{w}\right)}^{\alpha }\right]}^{\frac{\beta }{2}}}$$

(24) $$G^{\prime \prime }\left(w\right)=(G_{g}-G_{0})\frac{{\left(\frac{w}{w_{0}}\right)}^{\frac{\alpha \beta }{2}}\sin\left[\beta \theta \left(w\right)\right]}{{\left[{\left(\frac{w}{w_{0}}\right)}^{\alpha }+2\cos\left(\frac{\alpha \pi }{2}\right)+{\left(\frac{w_{0}}{w}\right)}^{\alpha }\right]}^{\frac{\beta }{2}}}$$

(25) $$\tan\left[\theta \left(w\right)\right]=\frac{\sin\left(\frac{\alpha \pi }{2}\right)}{\left[{\left(\frac{w}{w_{0}}\right)}^{\alpha }+\cos\left(\frac{\alpha \pi }{2}\right)\right]}$$

Similar to the previous results for the 2S2P1D model, the dynamic modulus and phase angle results expressed in terms of the MHN model can be obtained by combining the results of the storage modulus and loss modulus, where the dynamic modulus results are shown in Equation (26) and the phase angle results are shown in Equation (27).

(26) $$\begin{array}{l} \left|G^{*}\left(\omega \right)\right|=\sqrt{{\left[G^{\prime }\left(\omega \right)\right]}^{2}+{\left[G^{\prime \prime }\left(\omega \right)\right]}^{2}}=\\ \sqrt{{\left[G_{0}+(G_{g}-G_{0})\frac{{\left(\frac{w}{w_{0}}\right)}^{\frac{\alpha \beta }{2}}\cos\left[\beta \theta \left(w\right)\right]}{{\left[{\left(\frac{w}{w_{0}}\right)}^{\alpha }+2\cos\left(\frac{\alpha \pi }{2}\right)+{\left(\frac{w_{0}}{w}\right)}^{\alpha }\right]}^{\frac{\beta }{2}}}\right]}^{2}+{\left[(G_{g}-G_{0})\frac{{\left(\frac{w}{w_{0}}\right)}^{\frac{\alpha \beta }{2}}\sin\left[\beta \theta \left(w\right)\right]}{{\left[{\left(\frac{w}{w_{0}}\right)}^{\alpha }+2\cos\left(\frac{\alpha \pi }{2}\right)+{\left(\frac{w_{0}}{w}\right)}^{\alpha }\right]}^{\frac{\beta }{2}}}\right]}^{2}} \end{array}$$

(27) $$\delta =\arctan\left(\frac{G^{\prime \prime }\left(\omega \right)}{G^{\prime }\left(\omega \right)}\right)=\arctan\left(\frac{\left(G_{g}-G_{0}\right){\left(\frac{w}{w_{0}}\right)}^{\frac{\alpha \beta }{2}}\sin\left[\beta \theta \left(w\right)\right]}{G_{0}{\left[{\left(\frac{w}{w_{0}}\right)}^{\alpha }+2\cos\left(\frac{\alpha \pi }{2}\right)+{\left(\frac{w_{0}}{w}\right)}^{\alpha }\right]}^{\frac{\beta }{2}}+\left(G_{g}-G_{0}\right){\left(\frac{w}{w_{0}}\right)}^{\frac{\alpha \beta }{2}}\cos\left[\beta \theta \left(w\right)\right]}\right)$$

In order to obtain the model parameters, the parameter solution can be accomplished by constructing the error function $ef_{2}$ for the storage modulus and loss modulus, where the expression of the error function $ef_{2}$ is shown in Equation (28).

(28) $$ef_{2}=ef_{G^{\prime }}+ef_{G^{\prime \prime }}=\frac{1}{N}\sqrt{\sum_{i=1}^{N}{\left(\frac{{\left|G^{\prime }\left(\omega \right)\right|}_{m,i}-{\left|G^{\prime }\left(\omega \right)\right|}_{p,i}}{{\left|G^{\prime }\left(\omega \right)\right|}_{m,i}}\right)}^{2}}+\frac{1}{N}\sqrt{\sum_{i=1}^{N}{\left(\frac{{\left|G^{\prime \prime }\left(\omega \right)\right|}_{m,i}-{\left|G^{\prime \prime }\left(\omega \right)\right|}_{p,i}}{{\left|G^{\prime \prime }\left(\omega \right)\right|}_{m,i}}\right)}^{2}}$$

where: $ef_{2}$ is the error function of storage modulus and loss modulus, %; $ef_{G^{\prime }}$ is the error function of storage modulus, %; $ef_{G^{\prime \prime }}$ is the error function of loss modulus, %; N is the test sample point of storage modulus, which has the value of 30; $G^{\prime }{\left(\omega \right)}_{m,i}$ is the sample point of measured storage modulus, MPa; $G^{\prime }{\left(\omega \right)}_{p,i}$ is the sample point of storage modulus predicted from the MHN model, MPa; $G^{\prime \prime }{\left(\omega \right)}_{m,i}$ is the sample point of measured loss modulus, MPa; $G^{\prime \prime }{\left(\omega \right)}_{p,i}$ is the sample point of predicted from the MHN model, MPa.

Similar to the previous 2S2P1D model results, functional forms of storage modulus and loss modulus master curves of different steel slag-crumb rubber modified asphalt mixtures were constructed based on the MHN model. The fitting parameters of the model were calibrated by the least squares method using the WLF shifting technique, taking into account the measured results of storage modulus and loss modulus. Among them, the MHN model storage modulus master curve model has 5 unknown parameters ($G_{0}$, $G_{g}$, $w_{0}$, α, β), in addition to 2 shift factor parameters ($C_{1}$, $C_{2}$) need to be determined. All model parameters in Equations (9) and (10) can be solved by constructing and minimizing the error function $ef_{2}$ for the storage and loss modulus. Where the expression of the error function $ef_{2}$ is given in Equation (28). All model parameters and shift factor parameters can be obtained using the Solve function of Microsoft Excel. Finally, paralleling the 2S2P1D model framework, Table 8 consolidates all MHN parameter results (model parameters and shift factor parameters), demonstrating close agreement between our model fitting outcomes and those reported by Zhao [26] et al. Furthermore, Table 9 presents validation statistics associated with the model fitting process, all of which provide compelling evidence for the model’s reliability. Supplementary validation through Appendix A.2, Table A4 confirms that the goodness-of-fit criteria satisfy the “Excellent” classification standard.

## 5. Results and Discussion

### 5.1. Dynamic Mechanical Characterization of Steel Slag Asphalt Mixtures in Narrow Frequency and Temperature Domains

#### 5.1.1. Effect of Temperature on Dynamic Modulus

**Figure 3:**
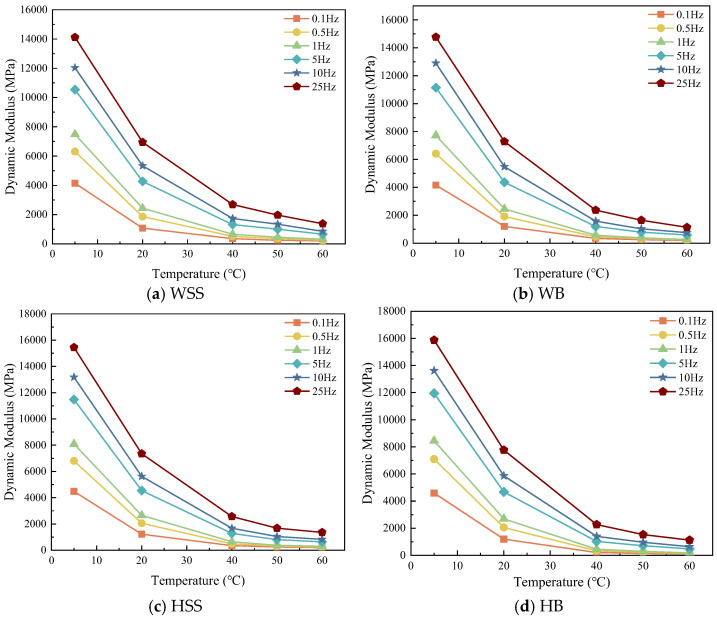
Dynamic modulus of steel slag-crumb rubber modified asphalt mixtures under varying temperatures: (**a**) WSS, (**b**) WB, (**c**) HSS, (**d**) HB.

The complex modulus of four steel slag asphalt mixtures (WSS, WB, HSS, and HB) was experimentally characterized under the test conditions specified in Section 2.2.3. The effect of temperature on dynamic modulus is shown in Figure 3.

Figure 3 demonstrate that under identical loading frequency conditions, the dynamic modulus of all steel slag-crumb rubber modified asphalt mixtures exhibits a temperature-dependent decrease. This indicates that the ability of the asphalt mixture to resist deformation at high temperatures also decreases, and its performance at high temperatures deteriorates. This is because the crumb rubber modified asphalt mixture is typical viscoelastic material, under high-temperature conditions, the asphalt binder softens, reducing the bonding capacity of the asphalt mixture, which leads to steel slag-crumb rubber modified asphalt mixture from the elasticity gradually changed to plasticity [27], the deformation of the load increases significantly resulting in decrease in dynamic modulus. In addition, the sharp reduction in dynamic modulus occurs when the temperature increases from 5 °C to 40 °C. This phenomenon is attributed to the softening of the asphalt binder, which affects its elastic recovery ability. Under the same loading conditions, the softened binder produces greater deformation, resulting in a significant decrease in dynamic modulus. However, as the temperature continues to increase (40 °C to 60 °C), the rate of decrease in dynamic modulus slows down. This is because at these higher temperatures, the viscosity of the asphalt binder has already reached the low threshold, and therefore further increases in temperature have less effect on the viscosity-dependent dynamic modulus behavior. Instead, the mechanical response are mainly influenced by the aggregate skeleton interlocking mechanism rather than the binder rheology.

#### 5.1.2. Effect of Frequency on Dynamic Modulus

**Figure 4:**
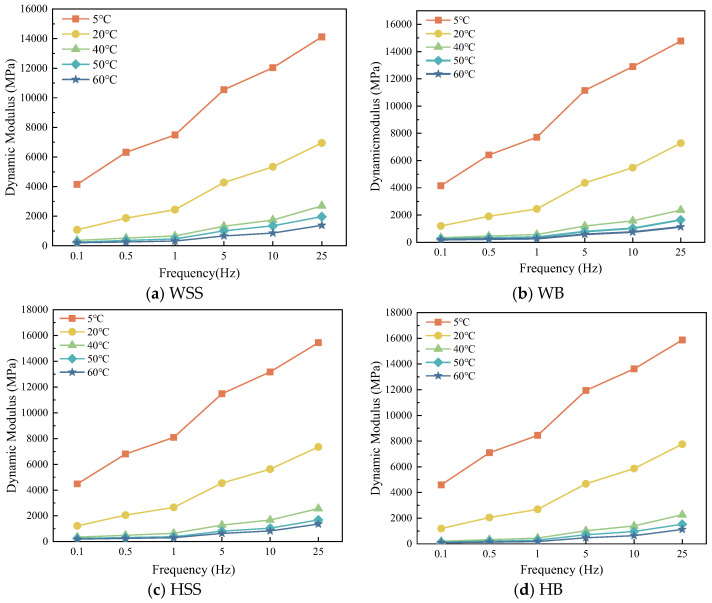
Dynamic modulus of steel slag-crumb rubber modified asphalt mixtures under varying frequencies: (**a**) WSS, (**b**) WB, (**c**) HSS, (**d**) HB.

The effect of frequency on dynamic modulus for the four types of steel slag asphalt mixtures is shown in Figure 4.

Figure 4 demonstrate that under isothermal conditions, the dynamic modulus of all steel slag-crumb rubber modified asphalt mixtures exhibits loading frequency-dependent enhancement, this is because at lower loading frequency polymeric chains within the binder undergo large-scale motions (such as molecular chain untangling, slip), resulting in viscous flow dominance and loss modulus dissipation [28]. With the increase of the loading frequency, the polymeric chains motion is gradually restricted, and when the loading frequency increases to certain degree allowing only small-scale local motion (e.g., bond angle vibration, side-base rotation), the material exhibits elastic response with significant enhancement of storage modulus, which is manifested as significant increase of the modulus. In addition, it can also be found that the dynamic modulus of asphalt mixtures is linearly and positively correlated with the loading frequency at the test temperatures of 5 °C and 20 °C, while the dynamic modulus of the four steel slag rubber powder modified asphalt mixtures is nonlinearly and positively correlated with the loading frequency at 40 °C, 50 °C, and 60 °C; in other words, the growth rate of the dynamic modulus increases with the increase of the loading frequency, which is attributed to the fact that the dynamic modulus is dependent on both the temperature and frequency.

#### 5.1.3. Comparison of Dynamic Modulus of Different Steel Slag Asphalt Mixtures in Narrow Frequency and Temperature Domains

**Figure 5:**
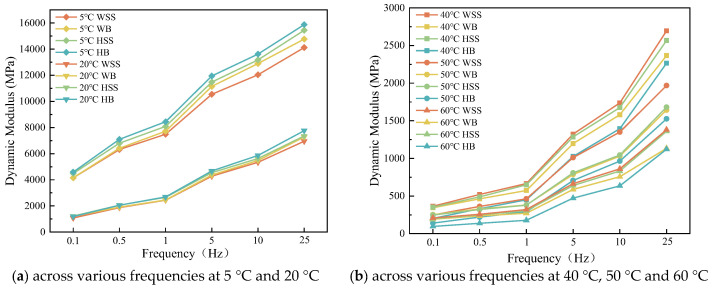
Comparison of dynamic modulus of different steel slag-crumb rubber modified asphalt mixtures under the same test conditions: (**a**) across various frequencies at 5 °C and 20 °C; (**b**) across various frequencies at 40 °C, 50 °C and 60 °C.

The dynamic modulus of the four steel slag asphalt mixtures under the same test conditions is shown in Figure 5.

Figure 5 demonstrates that under low-temperature/high-frequency conditions, all four types of steel slag asphalt mixtures exhibited significantly higher dynamic modulus values. Particularly under the combined conditions of 5 °C and 25 Hz, the mixture’s dynamic modulus reached as high as 14,000 MPa, indicating predominantly elastic behavior and superior resistance to deformation. Conversely, under high-temperature/low-frequency conditions, the dynamic modulus showed substantial reduction, with the mixture registering merely 100 MPa under the combined conditions of 60 °C and 0.1 Hz, reflecting a predominantly viscous response and diminished load-bearing capacity. The higher the high-temperature dynamic modulus of asphalt mixtures under identical loading conditions, the greater deformation resistance, and the lower permanent rutting deformation [29]. It can be seen that permanent deformation is most likely to occur under high-temperature and low-frequency conditions. Taking the results of 10 Hz as an example [30], the rate of change of dynamic modulus of steel slag-crumb rubber-modified asphalt mixtures at different temperatures before and after the addition of warm-mix additives was compared, and the results are shown in Table 10.

Table 10 demonstrates that the dynamic modulus of the steel slag-crumb rubber modified asphalt mixture exhibited contrasting responses to warm-mix additives in different temperature ranges. Specifically, the incorporation of warm-mix additives resulted in 5.2–8.7% reduction in dynamic modulus at lower service temperatures (5 °C and 20 °C). Conversely, at higher service temperatures (40 °C, 50 °C, and 60 °C), the additives enhanced the mixture’s dynamic modulus by 3.5–29.3%, showing significant temperature-dependent performance characteristics. This behavior suggests that the warm-mix additive increases the flexibility of asphalt mixture at low temperatures and improves the elasticity of asphalt mixture at high temperatures. This performance enhancement primarily from two synergistic mechanisms induced by warm-mix additives. Firstly, the reduced mixing temperature decreases thermal aging of the asphalt binder, thereby improving the mixture’s post-compaction flexibility and crack resistance. Secondly, the additives modified the asphalt’s molecular architecture through: (a) increased concentration of unsaturated carbon bonds (C≡C and C≡C); (b) optimized dispersion of asphaltenes within the colloidal structure. The homogenized asphaltene distribution facilitates alignment of long-chain hydrocarbons, which undergo crosslinking to establish an elastic polymer network. This microstructural reorganization enhances stress dissipation capacity at both binder and mixture scales, ultimately improving overall elasticity [31]. In addition, the SDYK surfactant molecule consists of hydrophilic groups (polar molecules) and lipophilic groups (non-polar molecules), in which the lipophilic groups can be bonded to the asphalt, however, the hydrophilic groups can be bonded to the aggregate. In other words, the addition of SDYK surfactant enhances the bond between asphalt and aggregate [32], which improves the deformation resistance of asphalt mixtures at high temperatures, and ultimately shows an increase in dynamic modulus.

In summary, under the same loading frequency and high-temperature conditions, the dynamic modulus of WSS is slightly larger than that of HSS, followed by WB, and the dynamic modulus of HB is the smallest. This indicates that compared with basalt-steel slag hybrid crumb rubber modified asphalt mixture, steel slag-crumb rubber modified asphalt mixture have good deformation resistance at high temperature, especially the warm-mixed steel slag-crumb rubber modified asphalt mixture have better performance at high temperature. In addition, under identical loading frequencies and low-temperature conditions, the dynamic modulus of HB is the largest, followed by HSS and WB, and the dynamic modulus of WSS is the smallest. This indicates that compared with the basalt-steel slag hybrid crumb rubber modified asphalt mixture, the steel slag-crumb rubber modified asphalt mixture has good low-temperature deformation resistance, and its accumulated temperature stress is smaller under the same conditions, and the low-temperature performance of the warm-mixed steel slag-crumb rubber modified asphalt mixture is optimal when the material strength is also same.

### 5.2. Dynamic Mechanical Characterization of Steel Slag Asphalt Mixture in Wide-Frequency and Temperature Domains

The dynamic mechanical properties of steel slag-crumb rubber modified asphalt mixtures before and after the addition of warm-mix additives were analyzed in both narrow temperature and frequency domains in Section 4.1, while the high and low temperature properties of four steel slag-crumb rubber modified asphalt mixtures are also investigated. The temperature of hot mix asphalt pavements undergoes significant variations throughout its lifecycle. During construction, the material temperature typically ranges from approximately 160 °C (rolling temperature) to 180 °C (mixing temperature). In service conditions, the pavements experience extreme thermal fluctuations, enduring winter temperatures as low as −40 °C in cold climates and summer surface temperatures reaching up to 60 °C in warm regions. Asphalt pavements are subjected to not only high-speed traffic loads but also thermal stress relaxation and accumulation during service. These combined loading conditions can persist for extended durations ranging from dozens of hours to even longer periods [33]. Given the extensive temperature and frequency ranges encountered in practical pavement applications, existing testing equipment proves incapable of operating effectively across such broad operational spectra. This technical limitation underscores the critical need for comprehensive investigation into the dynamic mechanical properties of steel slag-crumb rubber modified asphalt mixtures, particularly regarding their performance characteristics before and after warm-mix additive across wide-temperature and frequency domains. Based on complex modulus tests, this study established the 2S2P1D mechanical element model and the MHN mathematical model following LVE theory and the TTSP. The dynamic mechanical properties of steel slag-crumb rubber modified asphalt mixtures were systematically investigated across broad temperature and frequency domains through these dual modeling approaches.

#### 5.2.1. Measured and Predicted Results of Storage Modulus and Loss Modulus

**Figure 6:**
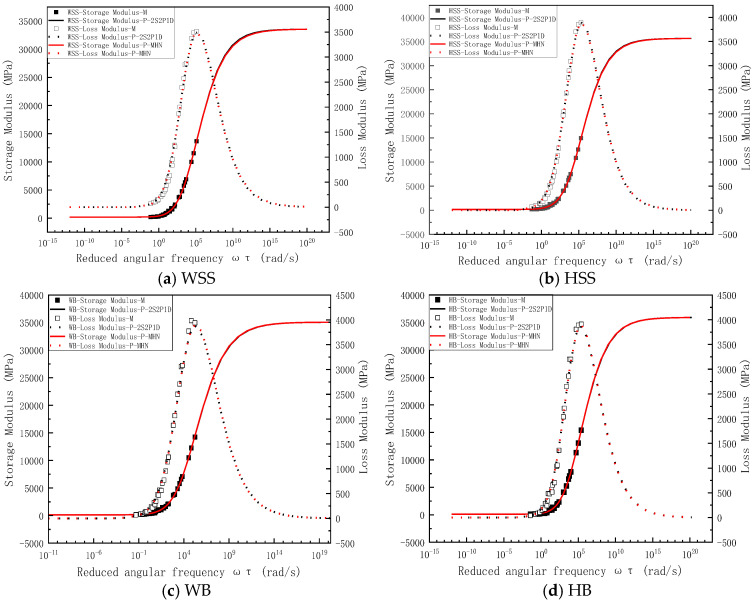
Measured and predicted results of storage and loss modulus for steel slag-crumb rubber modified asphalt mixtures: (**a**) WSS, (**b**) HSS, (**c**) WB, (**d**) HB.

To facilitate comparative analysis between measured and predicted results of storage modulus and loss modulus in steel slag asphalt mixtures, the results are presented in two complementary formats: (a) semi-logarithmic plots of reduced frequency relationships and (b) equality line plots of experimental versus predicted values. Figure 6a specifically demonstrates the correlation between measured and predicted storage/loss modulus for WSS mixtures, presented through semi-logarithmic coordinates of reduced frequency. As shown in Figure 6a, the predicted storage modulus and loss modulus derived from both the 2S2P1D model and the MHN model exhibit excellent agreement with the experimental measurements, indicating that the predictions from these distinct models demonstrate a high degree of consistency. Notably, the master curve of storage modulus exhibits a characteristic inverted Z-shaped profile. In other words, when the reduced frequency is extremely low, the steel slag asphalt mixtures exhibit rubbery state, characterized by minimal storage modulus of approximately 150 MPa, which remains constant regardless of frequency variations. Conversely, at high reduced frequencies, the storage modulus significantly increases to around 35 GPa, also maintaining stability across different frequencies, indicating glassy solid state of the steel slag asphalt mixture. In the intermediate range of reduced frequencies, the mixture demonstrates viscoelastic solid behavior, with the storage modulus showing a rapid increase in response to frequency elevation.

As illustrated in Figure 6a, the loss modulus master curve exhibits a bell-shaped profile, demonstrating distinct frequency-dependent behavior compared to the storage modulus. Under extremely low or high reduced-frequency conditions, the loss modulus approaches 0 MPa, corresponding to the material’s rubbery and glassy states, respectively, where stress and strain remain in phase. Within the intermediate reduced-frequency range, the asphalt mixture displays viscoelastic behavior: The loss modulus initially increases with rising reduced frequency, reaches its maximum value at the frequency corresponding to the most significant rate of storage modulus variation, and subsequently decreases with further frequency elevation, forming a characteristic loss modulus peak. Figure 6b–d present the experimental measurements and model predictions of the storage modulus and loss modulus for HSS, WB, and HB mixtures, respectively. As evident from these figures, similar to the WSS results, the model predictions for both the storage modulus and loss modulus closely align with the experimental measurements, with minimal discrepancies observed between different models. Additionally, the master curves of the storage modulus exhibit an inverted Z-shape, while those of the loss modulus display a bell-shaped profile. The interpretation principles underlying these curves are consistent with those applied to the WSS mixtures; thus, they are not reiterated here for brevity. Notably, the fundamental shapes and characteristic features of both storage and loss modulus master curves obtained in this study show complete consistency with the findings reported by Sun [34] thereby validating the reliability of our modeling results.

**Figure 7:**
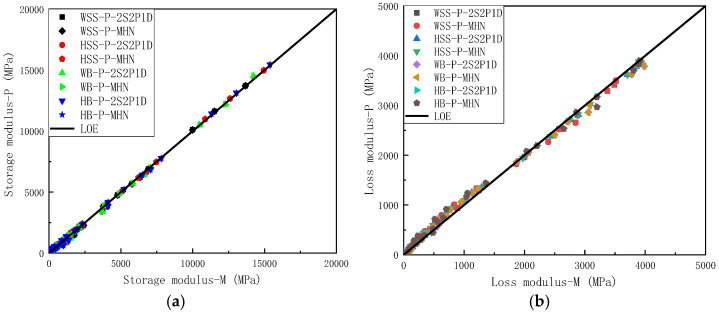
Equality line plots of storage modulus loss modulus for all asphalt mixtures: (**a**) storage modulus, (**b**) loss modulus.

To eliminate the frequency-dependent effects on both measured and predicted storage modulus and loss modulus, equality line plots were constructed for various mixtures by plotting experimentally measured storage modulus versus predicted storage modulus (Figure 7a) and applying the same methodology for loss modulus comparisons (Figure 7b). The equality line plots of storage modulus in Figure 7a reveals near-perfect alignment of experimental measurements with model predictions along the line of equality (LOE), a remarkable consistency observed in both 2S2P1D and MHN model simulations. This exceptional agreement not only demonstrates virtual equivalence between measured and predicted storage modulus but also indicates minimal discrepancies between predictions from different constitutive models. Figure 7b reveals similar consistency for loss modulus predictions, where data points from both models remain closely clustered around the LOE, and the predictions of the different models exhibit negligible differences. However, compared to the storage modulus results, the agreement shows a slight reduction in consistency. This disparity arises from the inherent prioritization of storage modulus accuracy during the least squares optimization process, a consequence of storage modulus values being typically an order of magnitude larger than loss modulus values. While the loss modulus predictions show marginally lower precision, the close proximity of experimental and modeled loss modulus values still confirms satisfactory predictive capability of both constitutive models.

In summary, Figure 6 and Figure 7 demonstrate that both the 2S2P1D and MHN models achieve near-perfect alignment between predicted and measured storage modulus and loss modulus values across all four asphalt mixtures, with negligible discrepancies observed between the two models’ predictions. This dual consistency not only confirms the superior predictive accuracy of the modeling frameworks but also reveals their functional equivalence in characterizing viscoelastic moduli, thereby validating their interchangeable application for asphalt mixture performance evaluation under varying loading conditions.

#### 5.2.2. Measured and Predicted Results for Dynamic Modulus and Phase Angle

**Figure 8:**
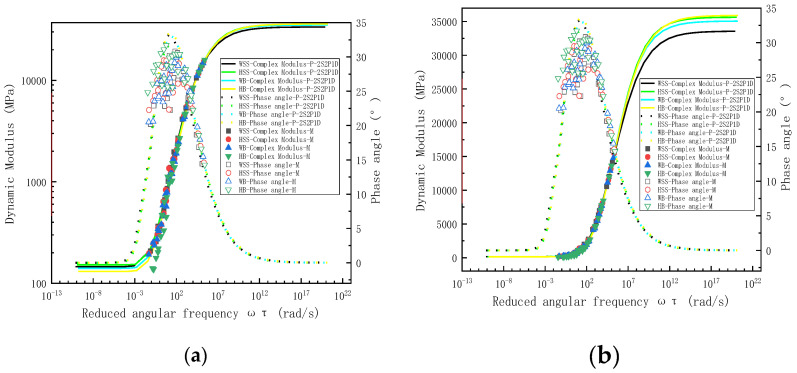
Measured dynamic modulus and phase angle of all asphalt mixtures and predicted results based on 2S2P1D modeling. (**a**) the bi-logarithmic coordinates of the modulus-reduced frequency, (**b**) the semi-logarithmic coordinates in reduced frequency.

**Figure 9:**
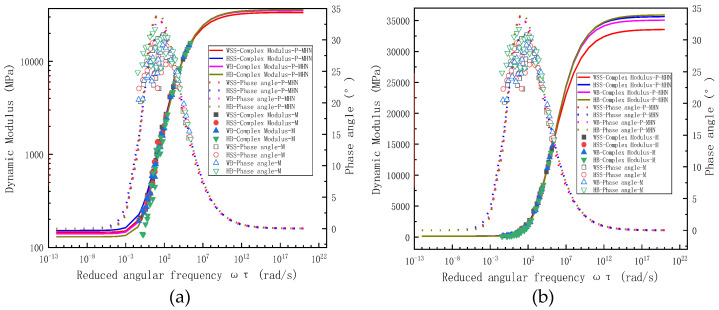
Measured dynamic modulus and phase angle of all asphalt mixtures and predicted results based on MHN modeling. (**a**) the bi-logarithmic coordinates of the modulus-reduced frequency, (**b**) the semi-logarithmic coordinates in reduced frequency.

Although previous investigations of steel slag asphalt mixtures have demonstrated the correlation between measured and predicted results through storage modulus and loss modulus representations, it should be emphasized that these viscoelastic parameters are fundamentally derived from dynamic modulus and phase angle measurements. To provide comprehensive validation of model fitting performance, Figure 8 present comparative analyses of experimental data and 2S2P1D model predictions using distinct visualization approaches: Figure 8a employs bi-logarithmic (log-log) coordinates to plot dynamic modulus against reduced frequency, while Figure 8b utilizes semi-logarithmic (log-linear) scaling for reduced frequency representation. Parallel evaluation of the MHN model is conducted in Figure 9, with Figure 9a illustrating dynamic modulus characteristics in bi-logarithmic coordinates and Figure 9b detailing phase angle behavior through semi-logarithmic representation, both maintaining consistent reduced frequency parameterization for direct comparison.

Figure 8 and Figure 9 reveal strong agreement between measured dynamic modulus values and predictions from both 2S2P1D and MHN models across the tested frequency spectrum. However, phase angle predictions exhibit frequency-dependent discrepancies—maintaining good correlation at medium–high frequencies (≥10^2^ rad/s) while deviating measurably at lower frequencies (<10^2^ rad/s). This discrepancy arises from two principal factors: First, the least-squares optimization inherently prioritizes accuracy in the medium–highigh-frequency range where storage and loss modulus demonstrate significantly greater magnitude (10^2^–10^4^ order difference), leading to amplitude-dependent weighting commonly termed the “large-value dominance” effect. Second, inherent measurement uncertainties in phase angle determination, derived from stress-strain hysteresis characterization, introduce systematic errors particularly pronounced at lower frequencies [35]. Notably, both constitutive models intrinsically satisfy the Kronig-Kramers (K-K) relations [36], theoretically enabling phase angle derivation from dynamic modulus data—an approach adopted by some researchers [37] to circumvent measurement limitations. The master curve analysis demonstrates fundamental viscoelastic consistency, with dynamic modulus and storage modulus master curves both exhibiting characteristic inverted Z-shapes, while phase angle and loss modulus master curves display complementary bell-shaped profiles. These morphological correlations fundamentally reflect the intrinsic TTSP of asphalt materials, confirming proper construction of the viscoelastic characterization framework [38].

**Figure 10:**
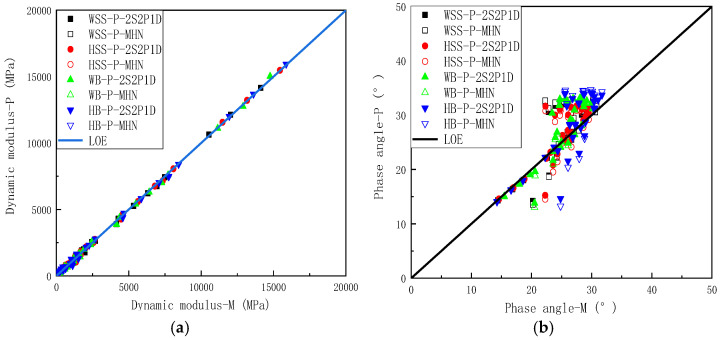
Equality line plots of dynamic modulus and phase angle for all asphalt mixtures; (**a**) dynamic modulus, (**b**) phase angle.

To mitigate the influence of frequency on dynamic modulus and phase angle, LOE analysis of dynamic modulus was conducted by plotting experimentally measured values on the abscissa against model predicted values on the ordinate. The equality line plots of dynamic modulus (Figure 10a) reveals near-perfect alignment between measured and predicted dynamic modulus values for both the 2S2P1D and MHN models, with minimal discrepancies observed between predictions from different models, confirming the models’ excellent fidelity across the full frequency spectrum. Analogous to the dynamic modulus analysis, LOE analysis of phase angle employed identical coordinate conventions to construct equality line plots for phase angle (Figure 10b). Figure 10b shows the equality line plots of phase angles, revealing that while the predicted phase angles from both 2S2P1D and MHN models do not perfectly align with experimental measurements, the model predictions generally lie on either side of the LOE with minimal discrepancies between different models. Although systematic deviations between model predicted and measured phase angles are observed, particularly within specific frequency ranges, this apparent discrepancy actually confirms the models’ robustness. Both formulations inherently satisfy Kramers-Kronig (K-K) relations through their constitutive frameworks. The preserved K-K relations compliance ensures theoretically consistent of phase angle predictions derived from dynamic modulus data, thereby exposing inherent limitations in experimental phase angle determination rather than model deficiencies. The observed prediction “inaccuracies” thus principally reflect measurement artifacts stemming from stress-strain hysteresis characterization challenges, particularly in low signal-to-noise regimes, rather than fundamental model shortcomings.

In summary, Figure 8 and Figure 9 demonstrate that both the 2S2P1D and MHN models achieve near-perfect agreement between predicted and measured dynamic modulus values across all four asphalt mixtures, with negligible discrepancies between the two models’ predictions. While phase angle predictions from both models also approximate experimental measurements, their accuracy is comparatively lower than that of dynamic modulus predictions, a discrepancy attributable to measurement distortions in phase angle values under high-temperature and low-frequency conditions rather than inherent model limitations. Notably, the 2S2P1D model exhibits superior phase angle prediction fidelity compared to the MHN model, as evidenced by its closer alignment with the LOE. This dual observation confirms the models’ high predictive accuracy while revealing their functional equivalence in dynamic modulus characterization. The statistical validation presented in Table 7 and Table 9 further substantiates that the models demonstrate comparable performance under standard conditions, yet prioritizes the 2S2P1D model for applications requiring stringent phase angle precision, particularly in scenarios involving complex viscoelastic responses.

#### 5.2.3. Comparison of Measured and Predicted Results in Cole–Cole Space and Black Space

**Figure 11:**
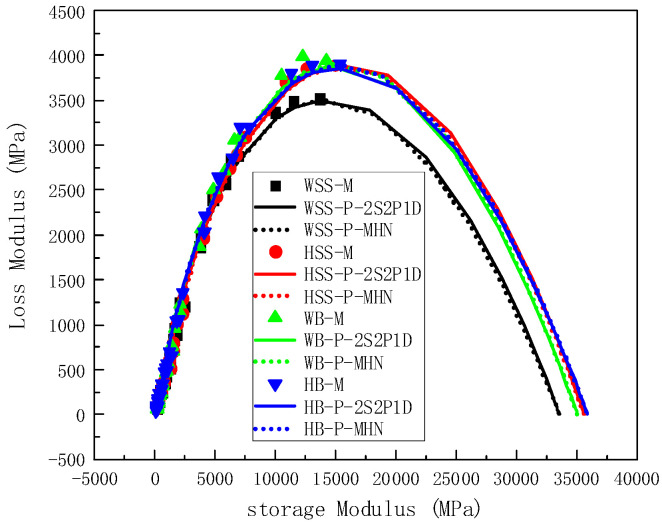
Cole–Cole plot of test results and predicted results.

**Figure 12:**
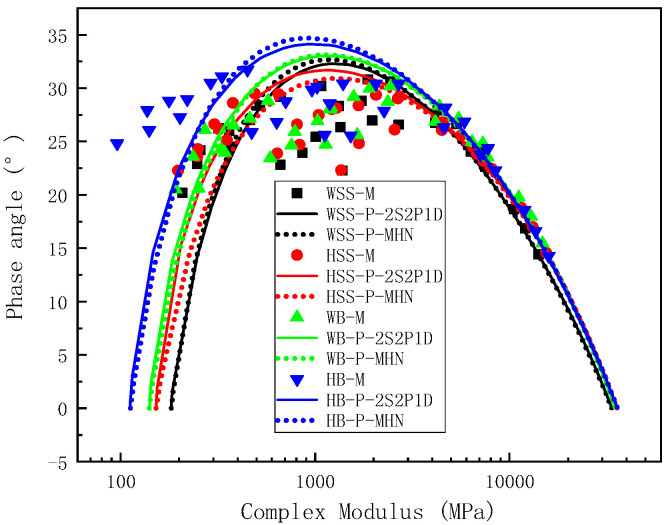
Black space plot of test results and predicted results.

In addition, Cole–Cole plots [39] and Black-space plots [40] are also used to assess the predictive reliability of both constitutive models. In particular, the Cole–Cole plot shows the relationship between storage modulus and loss modulus, while the Black-space plot shows the relationship between dynamic modulus and phase angle, as shown in Figure 11 and Figure 12 respectively. Both models demonstrate excellent agreement with experimental data in Cole–Cole space (Figure 11), with predicted curves faithfully tracking the characteristic trajectory of measured values. The observed $G^{\prime }$-$G^{\prime \prime }$ relationship reveals fundamental material behavior—as storage modulus increases, loss modulus initially rises then declines, corresponding to the anticipated non-monotonic phase angle evolution [41]. Analysis of minimum dynamic modulus predictions shows remarkable consistency between models, yielding values converge at 150 MPa across mixture formulations, in full accordance with master curve extrapolations. Similarly, maximum dynamic modulus predictions converge at 35 GPa, maintaining tight correlation with master curve-derived estimates despite minor formulation-dependent variations. This dual-model consensus across both extreme modulus values and characteristic curve morphology confirms robust predictive capability, while the observed formulation-specific variations fall well within acceptable thresholds for asphalt mixture characterization.

Figure 12 presents Black-space diagram comparisons of experimental and predicted viscoelastic parameters (dynamic modulus vs. phase angle) for both warm and hot steel slag asphalt mixtures using the 2S2P1D and MHN constitutive models. While systematic deviations between predictions and measurements are observable across the test range, these discrepancies primarily originate from experimental phase angle uncertainties inherent in stress-strain hysteresis characterization—a limitation clearly evidenced by the characteristic divergence patterns in Black-space morphology [42]. Crucially, both models maintain intrinsic compliance with the K-K relations through their mathematical formulations, ensuring theoretically consistent interparameter relationships that validate prediction reliability despite measurement artifacts. The predicted curves exhibit characteristic inverted U-shaped trajectories (“gate” morphology), accurately capturing the fundamental material response where phase angle initially increases then decreases with rising dynamic modulus. Both models successfully identify the critical inflection point marking maximum phase angle occurrence at specific dynamic modulus thresholds, consistent with master curve extrapolations. This predictive capability persists even at modulus reduction extremes, demonstrating robust numerical stability across the viscoelastic transition spectrum.

#### 5.2.4. Analysis the Dynamic Modulus and Phase Angle of Steel Slag Asphalt Mixtures in Wide Frequency and Temperature Domains

**Figure 13:**
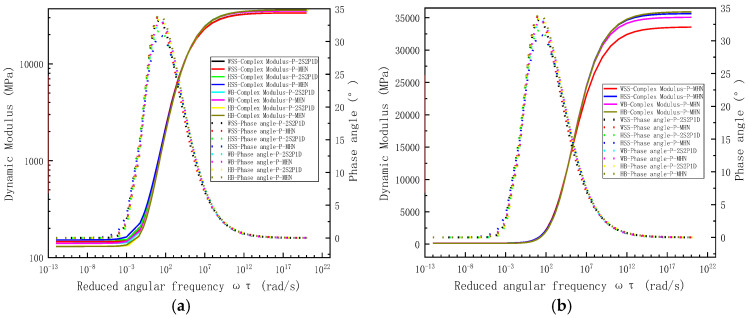
Analysis of dynamic modulus master curve and phase angle master curve results for steel slag asphalt mixtures: (**a**) the bi-logarithmic coordinates of the dynamic modulus-reduced frequency, (**b**) the semi-logarithmic coordinates in reduced frequency.

The previous analysis has validated the accuracy and reliability of the model. To further compare the dynamic mechanical properties of various warm-mix steel slag asphalt mixtures across a broad frequency range, the dynamic modulus and phase angle results were presented using bi-logarithmic plots of dynamic modulus versus reduced frequency and semi-logarithmic plots of phase angle versus reduced frequency, respectively. These results are illustrated in Figure 13. Examination of the dynamic modulus master curves in Figure 13 reveal a characteristic inverted Z-shape with increasing reduced frequency. At very low reduced frequencies, all mixtures exhibit rubbery state, characterized by a negligible and frequency-independent dynamic modulus. Conversely, at high reduced frequencies, the dynamic modulus stabilizes at a large, frequency-independent value, consistent with glassy solid behavior. In the intermediate reduced frequency range, the mixtures transition to a viscoelastic state, where the dynamic modulus increases sharply with frequency. Simultaneously, Figure 13 demonstrate that the phase angle master curve follows a distinct bell-shaped. At extreme reduced frequencies (very low or very high), the phase angle approaches 0°, corresponding to in-phase stress-strain relationships in the rubbery and glassy states, respectively. Within the intermediate frequency range, the phase angle initially increases with reduced frequency, reaches peak, and subsequently declines. Notably, the phase angle peak occurs at a lower reduced frequency than the loss modulus peak, the phenomenon attributable to the rapid increase in storage modulus within the frequency range where the phase angle peaks. This behavior underscores the dominance of elastic energy storage over viscous dissipation in this critical frequency regime [43].

**Figure 14:**
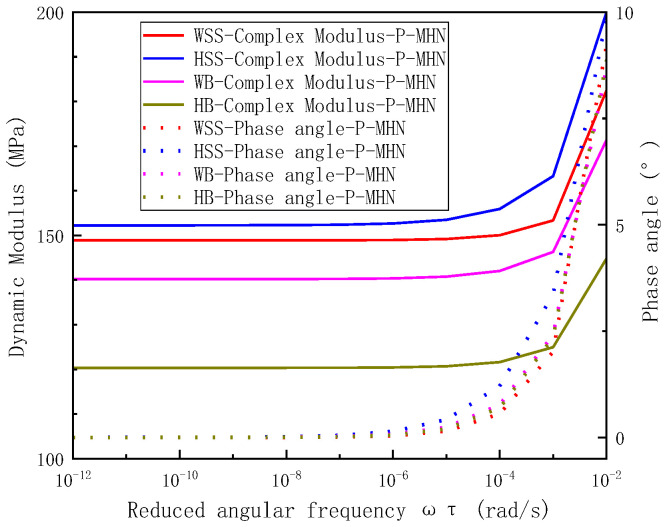
Localized enlargement of the reduced frequency from 10^−12^ to 10^−2^ in Figure 13a (the bi-logarithmic coordinates of the dynamic modulus-reduced frequency).

To enhance the clarity of the dynamic modulus and phase angle results at lower reduced frequencies, localized zoomed-in plots for reduced frequencies ranging from 10^−12^ to 10^−2^ rad/s are provided in Figure 14, complementing the data in Figure 13. Analysis of Figure 13a and Figure 14 reveals that the dynamic modulus of different steel slag asphalt mixtures decreases significantly at lower reduced frequencies (<10^−2^ rad/s). Specifically, the dynamic modulus of HSS is slightly higher than that of WSS, followed by WB, with HB exhibiting the lowest values. Concurrently, the phase angles of these mixtures remain relatively small and closely aligned, with HSS displaying the largest phase angle, followed by WB, HB, and WSS, which shows the smallest phase angle. As the reduced frequency decreases further (<10^−5^ rad/s), the dynamic modulus of all mixtures converges to smaller, stable values: 148.9 MPa for WSS, 152.2 MPa for HSS, 140.7 MPa for WB, and 121.0 MPa for HB. Simultaneously, the phase angles approach 0°. This behavior can be attributed to the dominant influence of aggregate properties on the dynamic modulus under very high temperatures or very low frequencies [44], where the gradation forms exhibit minimal variation despite slight differences in aggregate types.

**Figure 15:**
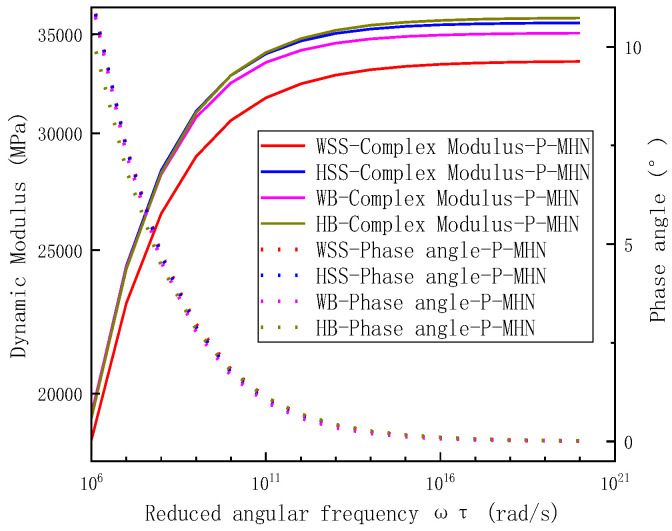
Localized enlargement of the reduced frequency from 10^6^ to 10^20^ in Figure 13b (the semi-logarithmic coordinates in reduced frequency).

To better visualize the dynamic modulus and phase angle behavior at elevated reduced frequencies, enlarged insets are provided in Figure 13b for the frequency range of 10^6^ to 10^20^ rad/s, with detailed representations shown in Figure 15. The analysis reveals distinct performance characteristics among steel slag asphalt mixtures under high reduced frequencies (>10^6^ rad/s). Specifically, the dynamic modulus demonstrates material-dependent variations where HB exhibits the highest values, followed sequentially by HSS, WB, and WSS. Concurrently, phase angles remain relatively low across all mixtures while maintaining discernible differences: WSS displays the largest phase angle, succeeded by HSS, WB, and HB in descending order. At extreme reduced frequencies (>10^15^ rad/s), the dynamic modulus stabilize at characteristic plateaus: 33.6 GPa (WSS), 35.6 GPa (HSS), 35.1 GPa (WB), and 35.9 GPa (HB), with phase angles asymptotically approaching 0°. This frequency-dependent behavior originates from the binder-dominated response regime under cryogenic conditions or ultra-high-frequency loading. Although identical asphalt binders were employed, differential interfacial adhesion characteristics emerge from variations in binder content and warm-mix additive formulations. These compositional differences alter the stress transfer efficiency at aggregate-binder interfaces, ultimately governing the observed mechanical responses.

The observed viscoelastic parameters (dynamic modulus and phase angle) of various steel slag asphalt mixtures exhibit comparable response patterns across intermediate reduced frequency ranges to those identified through narrow-frequency band analyses. Given this consistency in fundamental mechanical behavior between spectral domains, detailed discussion of intermediate-frequency characteristics is intentionally omitted to avoid redundant exposition while maintaining analytical focus on novel findings.

## 6. Conclusions

This study investigates the LVE properties of warm-mixed steel slag asphalt mixtures through complex modulus testing across multiple frequency domains. Narrow-frequency domain behavior was directly characterized using experimental complex modulus measurements, while wide-frequency domain analysis was achieved through LVE theory combined with TTSP. Given the multitude of available rheological models for asphalt mixture characterization, this research specifically employs the 2S2P1D model and the MHN model, selected for their inherent compliance with the K-K relations. The principal findings are summarized as follows:

1. The dynamic modulus of different types of steel slag asphalt mixtures increases continuously with decreasing temperature and increasing frequency in both narrow-frequency and temperature domains, which is determined by the viscoelasticity of the material. However, the dynamic modulus of different types of steel slag asphalt mixtures does not increase with decreasing temperature and increasing frequency in the same proportion. Under identical loading frequency and high-temperature conditions, WSS exhibits a slightly higher dynamic modulus than HSS, followed by WB, with HB demonstrating the lowest value. Conversely, under low temperature conditions with equivalent loading frequency, HB achieves the highest dynamic modulus, succeeded by HSS and WB, while WSS registers the smallest magnitude. This temperature-dependent inversion in viscoelastic performance hierarchy systematically reveals the material-specific thermorheological characteristics of the four asphalt mixtures, providing critical insights for pavement design optimization under varying climatic conditions.
2. Within the linear viscoelastic (LVE) range, the 2S2P1D and MHN models exhibit equivalent efficacy in characterizing the rheological behavior of steel slag-modified asphalt mixtures. Statistical analyses demonstrate negligible discrepancies between model-predicted viscoelastic parameters (dynamic modulus, storage modulus, loss modulus) and experimental measurements. Although minor deviations exist in phase angle predictions, these arise from inherent measurement errors of phase angles under high-temperature/low-frequency conditions rather than model deficiencies. Conversely, the intrinsic consistency of both 2S2P1D and MHN models with K-K relations fundamentally ensures predictive reliability. Provided that dynamic modulus test data maintain specified high-precision thresholds, these constitutive models manifest robust predictive capabilities across the full time temperature superposition spectrum. This mechanism originates from the intrinsic mathematical coupling between real and imaginary parameters inherent in K-K-compliant models.
3. Comprehensive analysis across extended frequency and temperature ranges reveals distinct master curve configurations for viscoelastic parameters. Both storage modulus and dynamic modulus master curves demonstrate characteristic inverted Z-shaped profiles, whereas phase angle and loss modulus master curves exhibit symmetrical bell-shaped distributions. This behavior indicates three distinct material states: (a) a rubbery-state response dominates under high-temperature or low-frequency conditions, (b) a glassy-state behavior prevails at low-temperature or high-frequency extremes, and (c) transitional viscoelastic-state characteristics emerge within intermediate temperature-frequency regimes. The observed master curve morphology fundamentally arises from TTSP governing steel slag-crumb rubber composite-modified asphalt mixtures rheological responses. In addition. when the reduced frequency (corresponding to high temperature and low frequency) is very low, the dynamic modulus of HSS is slightly larger than that of WSS, followed by WB, and the dynamic modulus of HB is the smallest. When the reduced frequency (corresponding to low temperature and high frequency) is high, the dynamic modulus of HB is slightly larger than that of HSS, followed by WB, and the dynamic modulus of WSS is the smallest.
4. The intrinsic measurement errors associated with phase angle determination fundamentally compromise the accuracy of narrow-frequency domain analyses for this parameter. Conversely, rheological modeling-based extrapolation enables reliable phase angle characterization across extended frequency ranges, particularly when employing theoretically consistent constitutive models. This predictive approach proves particularly valuable for addressing scenarios involving incomplete phase angle data or discontinuous measurement ranges, effectively complementing experimental limitations through physically meaningful interpolation.
5. This study investigates the dynamic mechanical characteristics of various warm-mixed steel slag-crumb rubber modified asphalt mixtures under narrow and broad frequency domains based on complex modulus test results, comparing their properties before and after warm-mixing. The findings provide critical references for formulating performance-based steel slag recycling specifications in green highway construction and offer technical support for large-scale implementation of warm-mixed steel slag modified asphalt mixtures. Furthermore, integrating the 2S2P1D or MHN model into mechanistic-empirical pavement design software could enhance the reliability of fatigue and thermal cracking predictions for these composite materials. It should be noted that the current research focuses exclusively on laboratory-based dynamic mechanical characterization under complex modulus conditions due to space limitations. Future studies should prioritize validation of long-term field performance under actual traffic loads and environmental conditions, incorporating real-time climatic data and traffic data to develop artificial intelligence-driven predictive models. Such advancements will bridge the gap between laboratory simulations and real-world engineering applications while optimizing sustainable pavement material design.

## Figures and Tables

**Table 1:** State of the art review on steel slag modified asphalt mixtures.

Author	Research Focus	Conclusions/Contributions
Shen Aiqin et al. [2]	Evaluate the shear deformation resistance and viscoelastic attenuation characteristics of fiber-reinforced steel slag asphalt mixtures	Incorporation of fibers enhances the dynamic modulus of the composites and the improvement is more pronounced in the low-frequency domain
Dinh et al. [3]	Evaluated the dynamic rutting resistance of steel wool fiber-modified asphalt mixtures	Steel wool fiber incorporation increased the dynamic modulus at high-service temperatures
Shiha et al. [4]	Evaluation of asphalt mixtures incorporating steel slag aggregates at varying substitution rates	With granular assemblies containing higher slag substitution rates exhibiting significantly enhanced load-bearing capacity and deformation resistance
Pathak et al. [5]	Investigation into the mechanical properties of open-graded asphalt mixtures incorporating steel slag aggregates at varying substitution ratios.	The integration of steel slag significantly enhanced the material’s resistance to rutting deformation, reduced susceptibility to cracking, and improved modulus characteristics
Zhao et al. [6]	Using steel slag and granite instead of conventional aggregates and compared them with asphalt mixtures prepared with limestone aggregates	Road performance of asphalt mixtures containing both granite and steel slag was improved
Ziaee et al. [7]	Investigation into the mechanical performance of warm-mix asphalt (WMA) formulations incorporating steel slag as coarse aggregate replacement	Significant improvements in mixture properties when steel slag was utilized
Cheng et al. [8]	Evaluation of warm-mix porous asphalt formulations incorporating steel slag aggregates and crumb rubber-SBS composite modified asphalt binders	Addition of warm-mix can significantly improve the low temperature crack resistance, and slightly reduce the water sensitivity, weaken the permeability, and have little effect on the modulus
Lee et al. [9]	Analysis of fatigue cracking characteristics and rutting behavior between two distinct steel slag-modified asphalt mixtures	Pavement systems incorporating steel slag aggregates exhibited superior rutting resistance compared to conventional Hot Mix Asphalt (HMA)
Wang et al. [10]	Quantified the skeleton structure of steel slag asphalt mixtures and investigated the enhancement mechanism of steel slag on the skeletal framework	The pronounced angularity of steel slag increases contact points between particles, while its coarse texture and non-spherical morphology enhance contact length
Zhang et al. [11]	Conducted a comparative analysis of interfacial adhesion performance across four material combinations: base asphalt-basalt, base asphalt-steel slag, rubber modified asphalt (RMA) -basalt and RMA-steel slag	The interfacial adhesion between steel slag and asphalt matrix significantly surpassed that of asphalt-basalt, with rubber modified asphalt demonstrating additional enhancing the adhesion with steel slag aggregates
Wei et al. [12]	Conducted a comprehensive investigation on the low-temperature performance of steel slag-crumb rubber modified asphalt mixtures	A positive correlation between steel slag content and enhanced low temperature performance: specifically, increased steel slag proportion significantly improved the mixture’s cracking resistance, creep deformation capacity, and stress relaxation efficiency
Lei et al. [13]	Investigated the interfacial transition zone (ITZ) characteristics of full proportion steel slag asphalt mixture (100SSA-AM), full proportion basalt asphalt mixture (100NCA-AM), and asphalt mixture of coarse aggregate steel slag and fine aggregate basalt (Hybrid-AM) using fractal dimension analysis	The basic oxygen furnace slag (BOFS) improved the roughness and structural complexity of ITZ, the Hybrid-AM exhibited 7.03% and 3.79% increases in ITZ fractal dimension compared to 100NCA-AM and 100SSA-AM, respectively

**Table 2:** Technical parameters of crumb rubber modified asphalt.

Technical Parameters	Experiment Result		Standard Value
	Crumb Rubber Modified Asphalt Binder	Warm Mix Crumb Rubber Modified Asphalt Binder	
Softening point/°C	66.4	68.7	≥55
Ductility @ 5 °C/cm	19.5	18.1	≥15
Penetration (25 °C, 100 g, 5 s)/0.1 mm	73.2	70.8	60~80
Rotational viscosity @ 175 °C/Pa·s	1.72	1.43	1~4
Difference in softening point @ 24 h and 135 °C/°C	1.8	1.7	≤3
RTFOT			
Residual penetration ratio @ 25 °C	82	80	≥60
Residual ductility @ 5 °C/cm	11.2	9.5	≥5
Mass change (no more than)/%	−0.5	−0.7	±1

**Table 3:** Technical parameters of warm-mix additive.

**Property**	**Experiment Result**	**Standard Value**
Appearance @ 25 °C	Yellow liquid	Yellow liquid
Viscosity @ 25 °C/mPa·s	650	500~1000
pH	12.0	11.5 ± 1
Amine/mg KOH/g	550	510~610

**Table 4:** Aggregate blend percentages.

Aggregate Size		10–20 mm	5–10 mm	3–5 mm	0–3 mm	Filler
Blend Percentage by Weight/%	steel slag	31%	19%	16%	31%	3%
	steel slag & basalt	33%	27%	9%	29%	2%

**Table 5:** Volume parameters of warm and hot mix steel slag-crumb rubber modified asphalt mixtures at optimum asphalt content.

Asphalt Mixture	Mix Temperature/°C	OAC/%	Gross Density g/cm^3^	Void/%	VMA/%	VFA/%	Stability/KN	Flow Value /mm
WSS	156	5.6	3.019	4.28	17.53	75.13	14.08	2.99
HSS	180	5.6	3.014	4.34	17.77	75.62	12.30	3.39
WB	156	5.4	2.838	3.67	14.82	76.89	12.88	2.57
HB	180	5.4	2.743	3.89	16.40	81.13	11.15	2.68

**Table 6:** 2S2P1D model fitting parameters for different types of steel slag-crumb rubber modified asphalt mixtures.

Asphalt Mixture	$E_{0}$	$E_{g}$	μ	$\tau_{0}$	k	h	β	$C_{1}$	$C_{2}$
WSS	145.89	33,572.8	2.30	1.27 × 10^−4^	0.22619	0.50951	3937.4	5.40	99.87
HSS	152.25	35,643.7	2.27	8.62 × 10^−5^	0.23888	0.48480	2094.1	7.63	120.63
WB	140.67	35,072.9	1.96	6.60 × 10^−5^	0.23240	0.49124	3466.3	8.44	132.39
HB	120.99	35,920.7	1.98	5.93 × 10^−5^	0.22401	0.49237	3145.9	10.22	146.35

**Table 7:** Test statistics for fitting results of different types of steel slag-crumb rubber modified asphalt mixtures based on the 2S2P1D model.

Asphalt Mixture	SSE	RMSE	r	R^2^	DC	χ^2^	F	*S*_e_/*S*_y_
WSS	6.09 × 10^5^	100.78	0.9990	0.9976	0.9963	134.94	3239.62	0.0710
HSS	5.48 × 10^5^	95.61	0.9993	0.9985	0.9979	181.86	3621.94	0.0539
WB	6.57 × 10^5^	104.66	0.9992	0.9982	0.9971	142.90	3111.87	0.0633
HB	7.97 × 10^5^	115.29	0.9989	0.9975	0.9969	327.52	2957.33	0.0654

where: Sum of Squares Error (SSE); Root Mean Square Error (RMSE); Correlation Coefficient (r); Adjusted R-squared (R^2^); Coefficient of Determination (DC); Chi-square Statistic (χ^2^); F-statistic (F); Standard Error of Estimation (*S_e_*); Standard Error of Deviation (*S_y_*).

**Table 8:** MHN model fitting parameters for different types of steel slag-crumb rubber modified asphalt mixtures.

Asphalt Mixture	$E_{0}$	$E_{g}$	$\omega_{0}$	α	β	$C_{1}$	$C_{2}$
WSS	145.89	33,572.8	1213.10	0.2102	2.8332	5.42	100.01
HSS	152.25	35,643.7	12,935.76	0.2309	2.0542	7.69	121.25
WB	140.67	35,072.9	7265.03	0.2319	2.2540	8.41	132.03
HB	120.99	35,920.7	4067.02	0.2211	2.5208	10.13	145.24

**Table 9:** Test statistics for fitting results of different types of steel slag-crumb rubber modified asphalt mixtures based on the MHN model.

Asphalt Mixture	SSE	RMSE	r	R^2^	DC	χ^2^	F	*S*_e_/*S*_y_
WSS	6.22 × 10^5^	101.82	0.9988	0.9973	0.9960	122.73	6688.14	0.0715
HSS	5.51 × 10^5^	95.78	0.9994	0.9985	0.9980	192.79	6978.47	0.0502
WB	6.67 × 10^5^	105.36	0.9991	0.9979	0.9970	136.24	6263.10	0.0615
HB	8.10 × 10^5^	116.19	0.9988	0.9973	0.9967	322.94	5934.36	0.0645

where: Sum of Squares Error (SSE); Root Mean Square Error (RMSE); Correlation Coefficient (r); Adjusted R-squared (R^2^); Coefficient of Determination (DC); Chi-square Statistic (χ^2^); F-statistic (F).

**Table 10:** Dynamic modulus change rate of steel slag-crumb rubber modified asphalt mixture before and after warm-mixing at 10 Hz.

Temperature/°C	Dynamic Modulus/MPa		Change Rate/%	Dynamic Modulus/MPa		Change Rate/%
	WSS	WSS		WB	HB	
5	12,033	13,174	−8.7	12,898	13,618	−5.3
20	5340	5630	−5.2	5478	5864	−6.6
40	1737	1675	3.7	1580	1394	13.3
50	1350	1044	29.3	1030	963	6.9
60	861	832	3.5	756	635	19.1

## Data Availability

The original contributions presented in this study are included in the article. Further inquiries can be directed to the corresponding authors.

## Abbreviations

The following abbreviations are used in this manuscript:

WSS	Warm-mix steel slag-crumb rubber modified asphalt mixture
HSS	Hot mix steel slag-crumb rubber modified asphalt mixture
WB	Warm-mix basalt-steel slag hybrid crumb rubber modified asphalt mixture
HB	Hot mix basalt-steel slag hybrid crumb rubber modified asphalt mixture
LVE	Linear viscoelastic
TTSP	Time–temperature superposition principle
2S2P1D	2 Springs, 2 Parabolic Elements and 1 Dashpot
MHN	Modified Havriliak–Negami
K-K	Kronig-Kramers
LOE	Line of equality
HN	Havriliak–Negami
WMA	Warm-mix asphalt
HMA	Hot mix asphalt

## Appendix A

### Appendix A.1

The technical specifications of steel slag coarse aggregate and basalt coarse aggregate are presented in Table A1 in the Appendix A.1, while those of steel slag fine aggregate and basalt fine aggregate can be found in Table A2 in the Appendix A.1. Limestone mineral powder was selected as the mineral filler, and its fundamental technical specifications are detailed in Table A3 in the Appendix A.1.

**Table A1:** Technical specifications of basalt coarse aggregate and steel slag coarse aggregate.

Items		Results						Standard Value
		Basalt			Steel Slag			
		3~5 mm	5~10 mm	10~20 mm	3~5 mm	5~10 mm	10~20 mm	
Apparent specific gravity		2.941	2.932	2.728	3.650	3.612	3.695	≥2.6
Saturated surface-dry bulk specific gravity		2.826	2.856	2.669	3.397	3.471	3.627	——
Bulk specific gravity		2.767	2.817	2.634	3.302	3.416	3.602	——
Water absorption/%		1.21	1.13	0.99	1.59	1.18	0.70	≤2.0
Ruggedness/%		3	3	2	1.4	1.3	0.9	≤12
Crushed stone value/%		——	——	16.7	——	——	11.6	≤26
Los Angeles abrasion value /%		——	10.3	10.3	——	8.1	8.1	≤28
Flat and elongated particles	Particle size > 9.5 mm/%	——	——	10.5	——	——	1.4	≤12
	Particle size < 9.5 mm/%	——	6.0	——	——	7.7	——	≤18
Chondrite content/%		1.8			0.2			≤3
Particle content < 0.075 mm by washing method/%>		0.5	0.5	0.8	——	——	——	≤1
Adhesion level		——			Level 4			≥Level 4
Free calcium oxide content/%		——			2.18			≤3

**Table A2:** Technical specifications of basalt fine aggregate and steel slag fine aggregate.

Items	Results		Standard Value
	Basalt	Steel Slag	
Apparent relative density	2.622	3.513	≥2.5
Clay content (<0.075 mm)/%	0.6	0.21	≤3
Sand equivalent value/%	85	68	≥60
Angularity/s	46.3	37.4	≥30

**Table A3:** Technical specifications of mineral filler.

**Items**		**Results**	**Standard Value**
Appearance		agglomeration-free	agglomeration-free
Apparent Density/(t/m^3^)		2.537	≥2.5
Sieves Size/%	<0.6 mm	100	100
	<0.15 mm	94.4	90~100
	<0.075 mm	75.7	75~100
Water Content/%		0.6	≤1
Hydrophilic Coefficient		0.5	<1
Plasticity Index/%		2.1	<4
Heating Stability		No change	Measured

### Appendix A.2

The validation statistics associated with model fitting results presented in Table 7 and Table 9 demonstrate high precision for both the 2S2P1D and MHN models. To further assess model accuracy, we employ the ratio of standard error to standard deviation (*S*_e_/*S*_y_) and the Adjusted R-squared (R^2^) to evaluate the goodness-of-fit between measured and predicted data. The corresponding goodness-of-fit evaluation criteria are detailed in Table A4 of Appendix A.2.

**Table A4:** Criteria of the goodness-of-fit statistics [45].

Criteria	Adjusted R-Squared (R^2^)	*S*e/*S*y
Excellent	≥0.90	≤0.35
Good	0.70–0.89	0.36–0.55
Fair	0.40–0.69	0.56–0.75
Poor	0.20–0.39	0.76–0.89
Very Poor	≤0.19	≥0.90
